# Spatial Patterns and Species Distribution Model-Based Conservation Priorities for *Scrophularia takesimensis* on Ulleungdo

**DOI:** 10.3390/plants14223498

**Published:** 2025-11-16

**Authors:** Gyeong-Yeon Lee, Na-Yeong Kim, Tae-Kyung Eom, Deokki Kim, Seung-Eun Lee, Tae-Bok Ryu

**Affiliations:** 1 Research Center for Endangered Species, National Institute of Ecology, Yeongyang 36561, Republic of Korea; ky5724@nie.re.kr (G.-Y.L.); xorud147@nie.re.kr (T.-K.E.);; 2 GFI Gochang Future Policy Institute, Gochang Food & Industry Institute, Gochang 56417, Republic of Korea; nayeong@gfi.re.kr

**Keywords:** endemic species, SDM, MaxEnt, microtopography, conservation prioritization, bias correction, time-series validation, Species Distribution Modeling

## Abstract

Conserving near-shore island endemics requires workflows that are robust to small, spatially clustered samples and that translate Species Distribution Model (SDM) into regulation-ready actions. We formalize a transferable SDM-to-action blueprint—(i) cluster-aware spatial holdout (leave-one-cluster-out, LOCO), (ii) conservative, high-specificity binarization paired with simple ecological filters, and (iii) explicit area-band uncertainty—and apply it to the Ulleungdo (Republic of Korea) endemic *Scrophularia takesimensis*. We combined 2008–2024 field records with a 5 m resolution MaxEnt model (linear–quadratic features; regularization RM = 1.40) using 28 unique presences versus 744 background points sampled within an accessible coastal belt (300 m from shore). Under LOCO, the model generalized well (AUC = 0.984 ± 0.014; partial AUC at specificity of at least 0.90 = 0.935; RelRMSE = 0.107) and mapped a narrow near-shore suitability belt with a continuous northern–northeastern core and fragmented southern–eastern satellites. To obtain a regulation-ready map, we converted continuous suitability to binary using a cutoff that achieved specificity of at least 0.98 under spatial holdout (threshold: 0.472; baseline: 300 m) and applied two ecological filters (retain areas within 90 m of shoreline; remove patches < 75 m^2^), yielding a CORE of 1.148 km^2^ that captured 71.4% of recent records with zero leakage beyond the belt after post-processing. Accessible-mask sensitivity (masks of 300, 450, and 600 m) bounded the post-processed CORE to 0.930–1.593 km^2^ (coverage: 0.607–0.789), which we carry forward as a planning area band. We translate these results into a tiered plan: protect the near-shore core, reconnect the fragmented southern and eastern stretches, and survey the highest-ranked coastal segments. Beyond this case, the blueprint generalizes to other small-n near-shore endemics, offering a transparent path from the SDM to policy while clarifying that, given static predictors, inferences concern present-day suitability rather than climate change forecasting.

## 1. Introduction

Island ecosystems are key reservoirs of biodiversity [1], but, owing to their isolation, are especially vulnerable to climate change and human disturbance [2]. Endemic island species face elevated extinction risks because of their restricted ranges and small population sizes; consequently, quantifying their current status and developing effective conservation strategies remain central tasks for global biodiversity conservation [3]. In Korea, the Ulleungdo endemic *Scrophularia takesimensis* Nakai epitomizes the urgency and importance of conserving island endemics.

*Scrophularia takesimensis* belongs to the family Scrophulariaceae within the genus *Scrophularia*. Roots of congeners (e.g., *Scrophularia striata*, *Scrophularia ningpoensis*) have long been used as the traditional materia medica Scrophulariae Radix, which is rich in iridoid glycosides such as harpagoside. Recent pharmacognostic studies have begun to examine *S. takesimensis* itself and report experimental anti-inflammatory activity, underscoring its cultural and biomedical relevance in addition to its conservation importance [4,5,6].

On Ulleungdo, *Scrophularia takesimensis* has been legally protected since 1993 (listed as a Protected Wild Species) and was classified as Endangered Wildlife Class II in 2022 [7]; as of 2025, *S. takesimensis* remains in Endangered Wildlife Class II under the Wildlife Protection and Management Act, which prohibits its capture, collection, possession, transport, and trade without permit, with criminal penalties. *S. takesimensis* is nationally categorized as Critically Endangered (CR) on the Korean Red List, assessed under the IUCN Red List Categories and Criteria v3.1 (Second edition) and applied at the national scale following the IUCN regional and national guidelines. The formal basis is B1ab(v)c(iv) + B2ab(v)c(iv), that is, very restricted EOO and AOO with continuing decline and extreme fluctuations in the number of mature individuals [8]. The species has not yet been globally assessed on the IUCN Red List (global category: Not Evaluated, NE) [9].

The species was first described by Nakai [10] and is known to occur around Sadong, Dodong, and other parts of Ulleungdo, with the exception of some southwestern areas [11,12]. It inhabits coastal forest edges, stone embankments, and gravel beaches [13]. Soils tend to be base-rich due to marine influence [12]. Populations near coastal roads face major threats, including habitat degradation from road expansion and tourism development, competitive suppression by other plants, wave action, and vegetation clearing [8,13]. Between 2011 and 2018, a resurvey of the original 39 sites documented loss of occupancy at 26 sites and a reduction in abundance from 430 to 98 individuals (an approximately 77% decline) [14]. Consistent with this biogeographic setting, phylogenetic analyses indicate that *S. takesimensis* descends from two ancestral lineages distributed in Japan and Sakhalin and subsequently diverged in situ on Ulleungdo [15].

Research on *Scrophularia takesimensis* spans its distribution and spatial pattern [11,13,16,17,18,19], vegetation and soils [12], propagation [20,21], and phylogeny and origin [15]. However, since 2020, there has been a shortage of studies that update its distributional status and leverage Species Distribution Models (SDMs) for management and conservation planning. Recent large-scale developments—such as the planned Ulleung airport and upgrades to coastal roads—pose direct risks to habitats and may exacerbate secondary impacts (e.g., slope failures during increasingly frequent extreme rainfall events under climate change [22]). Against this backdrop of accelerating and interacting threats, it is urgent to quantify recent distributional change and set data-driven conservation priorities for the species. While earlier studies mainly provided one-time status maps or at most two-time comparisons for *S. takesimensis* [11,13,16,17,18,19], there has been sparse work since 2020 that couples island-wide field data with a management-ready SDM.

Species distribution models provide a principled framework to relate occurrences to environmental gradients for explanation and prediction, and they are particularly useful for island floras where access is difficult and sample sizes are small. For near-shore endemics, fine-resolution topographic and coastal-exposure proxies can outperform coarse bioclimatic layers, and SDMs have informed conservation planning and targeted surveys for multiple insular taxa [23,24]. Within SDMs, MaxEnt is a presence–background algorithm that estimates a maximum-entropy distribution subject to environmental constraints [25,26]; when model complexity is controlled and validation is spatially explicit, it performs well with small-n presence-only data. Practical guidance emphasizes modest feature sets and adequate regularization to reduce overfitting [27,28,29]. Although ensemble SDMs are often recommended to bracket algorithmic uncertainty, we chose MaxEnt in this study because our dataset comprises a small number of spatially clustered presences, typical of protected island endemics. In addition, we address its common pitfalls by using cluster-based spatial cross-validation, conservative lq features with tuned regularization (RM = 1.40), and explicit uncertainty bracketing via accessible background sensitivity and thresholding and post-processing, retaining interpretability while capturing much of the robustness that ensembles seek to provide.

Here, we combine long-term national field surveys of endangered wildlife (2008–2024) with an SDM to address three questions: (i) How has the spatial distribution of *S. takesimensis* changed from 2008 to 2024? (ii) Which environmental factors currently structure its distribution, and where are the potential habitats located? (iii) How can these results be translated into spatial priorities for the conservation, restoration, and targeted discovery of new localities? By answering these questions, our study aims to provide directly actionable, science-based guidance for policy and on-the-ground conservation of a nationally endangered island endemic.

## 2. Materials and Methods

### 2.1. Study Area

Ulleungdo is a volcanic island at the eastern end of the East Sea, Korea (area 72.9 km^2^; shoreline 64.43 km) [30]. The island formed from the late Pliocene to the Holocene [31]. The Nari caldera occupies the center, and cone-shaped highlands > 900 m a.s.l. surround Seonginbong (984 m). Bedrock is dominated by trachyte, with reported high-alkali signatures [31,32]. Steep slope and dissected coastal landforms create sharp microclimatic gradients through aspect, insolation, and wind exposure. The climate is oceanic; in 2001–2010, the mean annual temperature was 13.7 °C (vs. 11.4 °C in inland Gyeongsangbuk-do) and mean annual precipitation 1461.7 mm (vs. 1291.6 mm) [33]. Seasonal precipitation contrasts are muted relative to the mainland, supporting vegetation where evergreen and warm-temperate elements coexist; numerous rare and endemic plants have been reported [34,35].

### 2.2. Data Sources and Pre-Processing

We compiled field records from the national Endangered Wildlife Nationwide Distribution Survey (2008, 2014, 2016, 2020, 2022, 2023, 2024). From each record, we extracted detection status (presence if count > 0; non-detection if count = 0), survey date, and GPS coordinates (latitude and longitude; WGS 84, decimal degrees). Eight records outside the administrative boundary were corrected using original site maps and address notes. Occurrence tables were imported with *readxl::read_excel* and cleaned using *dplyr::mutate/select/distinct*. Features were georeferenced as WGS84 points and transformed to UTM Zone 52N using *sf::st_as_sf* and *sf::st_transform* (EPSG:32652), then clipped to the island boundary with *sf::st_intersection*. Raster alignment and masking used *terra::project*, *terra::resample* (method = “bilinear” for continuous; “nearest” for categorical), *terra::crop*, and *terra::mask*.

Because *S. takesimensis* is nationally endangered and vulnerable to poaching and habitat damage, precise coordinates are withheld; outputs are summarized on a 500 m grid for de-identification.

### 2.3. Periodization and Definition of Unique Sites

To match the 5-year national review cycle, we defined four periods: (1) 2008, (2) 2014–2016, (3) 2020–2022, and (4) 2023–2024. To avoid pseudo-replication, occurrences were grouped to site-representative points using DBSCAN (*dbscan::dbscan*, ε = 30 m, minPts = 1, [36]), which merges duplicates and nearby detections within typical clump sizes of *S. takesimensis* (20–30 m [16]) and plausible GPS error in steep coastal terrain (10–30 m). Sensitivity checks at ε = 20 and 40 m followed the same workflow. For each period, we computed the number of unique sites and detection rates (presence sites divided by the total number of surveyed sites) with 95% bootstrap CIs using *boot::boot* (R = 1000). The 500 m analysis grid was created with *sf::st_make_grid* (cellsize = 500) and populated by spatial join (*sf::st_join*).

### 2.4. Grid-Based Change Classification

A 500 m grid was overlaid on Ulleungdo. Pooling all records, we dichotomized detections into past (2008–2022) and recent (2023–2024) and classified each cell as persistence (detected in both periods), loss (detected only in the past), gain (detected only in the recent period), or no detection (absent in both).

### 2.5. Species Distribution Modeling

We fitted a presence–background MaxEnt model using maxnet [37], implementing the MaxEnt algorithm [25,26]. Predictions were produced on the cloglog scale. Predictor variables were derived from a 5 m DEM [38] to capture topography, slope, relief, and coastal proximity, prioritizing temporally stable layers: elevation (ELEV_M), slope (SLOPE_DEG), terrain ruggedness index (TRI), terrain position index (TPI; circular window radius approximately 90 m at 5 m resolution, then resampled), aspect components (EASTNESS, NORTHNESS), Euclidean distance to shoreline (DIST_COAST_M), heat-load index (HEAT_LOAD), an approximate wetness index derived from local relief and slope (our implementation uses a TWI proxy computed as the sum of (−relief) and (−slope), consistent with the code), wave exposure (WAVE_EXP), and coastal and cliff metrics (EDGE_SLOPE, CLIFF_NEAR). The shoreline was derived from the island administrative boundary; distances were computed as Euclidean distances in UTM Zone 52N at 5 m resolution. All predictor rasters were aggregated to 90 m for model fitting and the trained model was then projected back to the native 5 m stack for mapping and post-processing. All patch-based rules (e.g., MMU = 3 cells = 75 m^2^) and the calculations for area and leakage were computed on the 5 m grid in EPSG:32652. Derived variables followed standard formulations: EASTNESS = sin(aspect) and NORTHNESS = cos(aspect) (aspect in radians); TPI used a circular window of approximately 90 m; HEAT_LOAD followed a McCune-type solar load using island latitude; the TWI proxy follows our relief plus slope approximation rather than the classic ln(a/tan β) form; coastal exposure (WAVE_EXP) and metrics for edges and cliffs (EDGE_SLOPE, CLIFF_NEAR) were derived from the 5 m DEM and shoreline geometry. Full parameter details are provided in Code S1 (Supplementary Materials).

We intentionally restricted predictors to topographic and coastal exposure variables. Ulleungdo’s small spatial extent strongly limits macroclimatic variation at the grid scales typically available for Korea (at least 1 km), and fine-scale topography is a better proxy for boundary-layer microclimate near the shore (e.g., salt-spray flux, humidity, temperature buffering). Accordingly, we treat DIST_COAST_M and ELEV_M not as isolated drivers, but as composite proxies for maritime exposure and unmeasured abiotic conditions at the coastal–forest ecotone. Because the predictors are essentially static, our model is not designed for forecasting under future climate scenarios; we therefore refrain from any temporal projections and interpret predictions as present-day suitability only.

The accessible background comprised valid 5 m cells within at most 300 m of the shoreline. We targeted a background:presence ratio of approximately 25:1 using simple random sampling without replacement (set.seed(42)). With 28 presence records, the realized ratio was 744/28 = 26.6:1. The accessible mask excluded NA across predictors, and for model-selection runs, a single background sample was drawn once and held fixed (set.seed(42)) to ensure reproducibility. To assess sensitivity to background intensity and the accessible mask, we refit the model across ratios from 10:1 to 30:1 (the 300 m mask) and additionally expanded the mask to 450 m and 600 m under the same design. These auxiliary runs used 5-fold cross-validation (single repeat) and are not directly comparable to LOCO. Predictive performance and management-oriented pre-policy areas were stable (mean AUC approximately 0.923–0.925; pAUC approximately 0.802–0.810; predicted area approximately 0.018–0.024 of the island’s area, i.e., approximately 1.3–1.8 km^2^), and the northern core axis persisted, indicating that conclusions are not sensitive to the exact realized ratio (26.6:1) relative to the target (approximately 25:1). We report pAUC at high specificity (*partial.auc = c(1, 0.90)*, *partial.auc.focus = “specificity”*, scale-correction enabled) to emphasize performance in low-false-positive regimes. To justify the 300 m accessible background, we relied on three lines of evidence: (i) the Q90 of 2020–2024 presences lies at approximately 90 m from the shoreline, indicating a strong coastal concentration; (ii) survey access and major disturbance processes (roads, revetments, spray) are largely confined to a few hundred meters inland; and (iii) when the mask is widened to 450–600 m, added terrain contributes negligible occupancy signals relative to the main coastal–forest ecotone. Therefore, we treat wider masks as stress tests rather than competing ecological hypotheses.

Before fitting, we reduced collinearity by removing pairs with Spearman |ρ| > 0.7 followed by iterative VIF pruning to no greater than 5 while protecting ecologically prioritized variables; the final retained set used in sensitivity checks is reported in Code S1 (Supplementary Materials). We evaluated feature sets {l, lq, lqh} and regularization multipliers (RM = 1.0–2.0 in 0.2 increments) on a grid. For each class × RM combination, we used 5-fold cross-validation to compute fold-wise AUC mean and SD, and selected models by maximizing a robustness score (mean AUC − 0.5 × SD). ΔAICc was computed from full-data fits for comparability across feature classes. The selected configuration (lq, RM = 1.40) lay within 2 ΔAICc units of the best per-class models, providing a parsimonious alternative to higher-order feature sets [27,28,29]. Hinge terms beyond lq were not adopted because they yielded minimal ΔAICc gains and lower stability at our sample size (n = 28), consistent with overfitting risks in small-sample presence–background settings.

Spatial external validation (LOCO): presence coordinates were clustered by DBSCAN (ε = 250 m, minPts = 1, [36]), and models were evaluated under leave-one-cluster-out at the cluster level. We chose ε = 250 m to aggregate proximate detections into ecological patches, exceeding the 30 m within-site spacing and approximating several canopy-height pixels and the 90 m coastal belt used for post-processing; this reduces residual spatial dependence and optimistic error estimates. In LOCO, clusters were held out at the presence-cluster level; evaluation used background samples from the accessible mask while excluding test-fold presence pixels. Partial AUC followed the same high-specificity setting (*partial.auc = c(1, 0.90)*). Sensitivity checks varying ε = 150–300 m produced similar out-of-fold performance (AUC 0.984–0.988; pAUC 0.931–0.939; Table A1).

**Figure A1:**
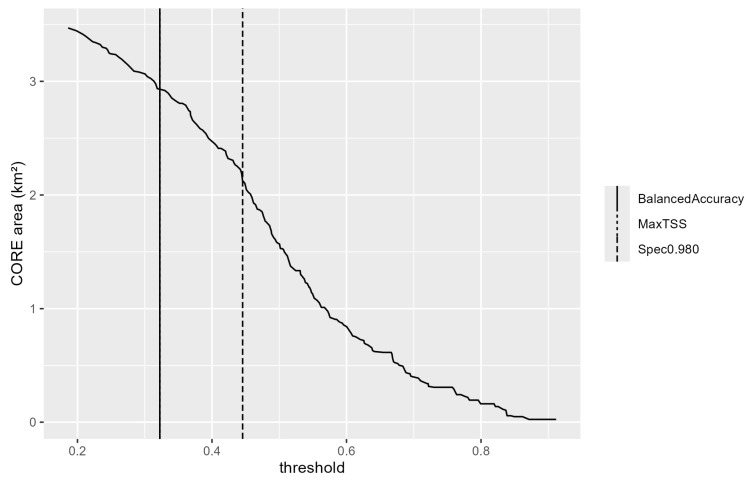
Threshold–area and threshold–coverage curves, with vertical lines showing thresholds chosen by Spec0.980 (dotted), MaxTSS (dashed), and Balanced Accuracy (solid; identical to MaxTSS here). Vertical lines mark the chosen thresholds. Area and coverage are post-processed (belt = 90 m, MMU = 3), whereas the ‘leakage’ values reported in the text refer to pre-belt areas.

For management decisions, we compared binarization rules on cloglog scores: Balanced-CV (median Youden-optimal across folds; used for tuning), TP10 (10th percentile of presence predictions), Coverage-target (minimum threshold achieving at least 0.90 presence coverage inside the belt), and Spec0.980 (98th percentile of background predictions). For single full-data summaries, we report Balanced (apparent) separately to avoid confusion with Balanced-CV. We also report MaxTSS (Youden’s J) and maximum balanced accuracy (BA) for comparability. The headline Spec0.980 threshold (0.472 in the LOCO baseline 300 m run) refers to the baseline accessible mask. In the separate ratio/mask sweep with new background draws (random CV), the 300 m case yielded 0.494, reflecting resampling variability. In addition to Spec0.980 for regulation-ready mapping, we provide MaxTSS and BA layers to guide survey expansion; comparative trade-offs (recall, mapped area, and off-belt leakage) are summarized in the Results and Figure A1, rather than being enumerated here. Candidate CORE cells satisfied all of the following: (i) cloglog at or above the chosen threshold, (ii) distance to coast at or below the 0.90 quantile (Q90) of 2020–2024 presence coastal distances (approximately 90 m), and (iii) contiguous patch size at least 3 cells (75 m^2^), using 8-neighbor connectivity. Spec0.980 thresholds were computed as the 0.98th quantile of background predictions from the exact training background sample in each run; numeric values therefore differ across accessible-mask sensitivity runs. Leakage was defined as the area with predictions at or above the chosen threshold located outside the coastal belt; areas were computed from 5 m pixels in EPSG:32652. Because belt clipping is a post-processing ecological filter, any statement of ‘no leakage beyond the 90 m belt’ refers to the processed product and must not be interpreted as empirical absence outside the belt.

**Figure A2:**
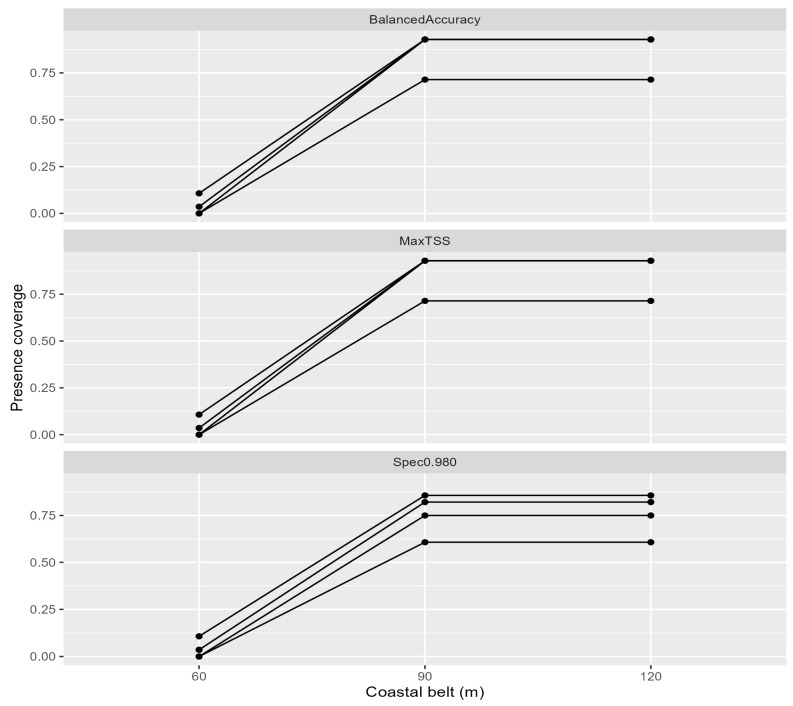
Coverage versus coastal-belt width and MMU. CORE panels are after belt clipping; the leakage panel shows pre-belt predictions outside the belt. Adopted setting: 90 m belt, MMU 3 cells. ‘No leakage’ refers to the post-processed product.

To verify that the conclusions were not artifacts of post-processing, we recomputed cores across coastal belts of 60, 90, and 120 m and MMU of 1, 3, 5, and 9 cells for Spec0.980, MaxTSS, and BA (Figure A2). The results show the following: (i) a negligible change from 90 → 120 m, (ii) sharp area and coverage losses at 60 m (frequent truncation of near-shore fragments), and (iii) predictable area decreases as MMU increased (e.g., Spec0.980 with 90 m belt: 2.44, 2.13, 1.65, 1.13 km^2^ for MMU = 1, 3, 5, 9 cells), while leakage outside the belt fell steeply from 60 → 90 m and then stabilized. Accordingly, we retained a 90 m belt and MMU = 3 cells (75 m^2^) as a conservative yet non-degenerate compromise, aligned with recent detections. Relative RMSE (RelRMSE; root mean square error) was computed as RMSE normalized by the range of observed presence and background scores within each validation fold.

Survey segment scoring used coastline segmentation with rolling medians on cloglog predictions (*terra::focal*) and rank adjustment by effort and safety priors (see Supplementary Materials (Code S1)). Performance metrics were computed with *pROC* (R) using *pROC::roc* and *pROC::auc* with *partial.auc = c(1, 0.90)*, *partial.auc.focus = specificity* and scale correction enabled.

### 2.6. Statistical Analyses and Software

All analyses were conducted in R 4.3.3 [39]. Raster handling and spatial operations were implemented entirely in R with *terra* [40]; vector handling: *sf* [41,42]; data wrangling: *dplyr* [43], *tidyr* [44], *purrr* [45], *readxl* [46]; modeling: *maxnet* [37]; performance: *pROC* [47]; visualization: *ggplot2* [48] (and tidyterra for map display [49]). All spatial operations, area computations, and patch filtering (8-neighbor connectivity) used EPSG:32652 with the random seed fixed at 42 for reproducibility. A complete, version-locked environment (renv.lock) and runnable scripts and parameter files are provided in Code S1 (Supplementary Materials).

## 3. Results

### 3.1. Number of Scrophularia takesimensis Occurrence Sites by Period

**Figure 1:**
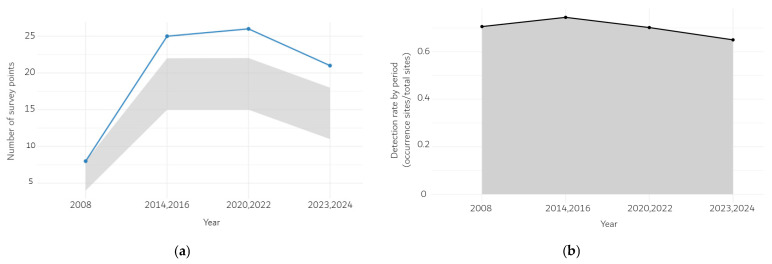
(**a**) Unique sites per period (DBSCAN ε = 30 m) with bootstrap 95% CIs; (**b**) detection rate (presence sites divided by total surveyed sites) with bootstrap 95% CIs. Gray ribbons denote 95% CIs (R = 1000 bootstrap replicates). Original coordinates withheld; summaries de-identified on a 500 m grid.

Using DBSCAN (ε = 30 m, minPts = 1) to spatially consolidate records, the numbers of unique *Scrophularia takesimensis* sites were 8 in 2008, 25 in 2014–2016, 26 in 2020–2022, and 21 in 2023–2024 (Figure 1a). After increasing through 2020–2022, the count declined to 21 in the most recent period (2023–2024), a 19.2% decrease from the peak (from 26 to 21).

Despite these changes, period-specific detection rates (occurrence sites divided by the total number of surveyed sites) remained stable between 0.65 and 0.75 across all periods (Figure 1b). Sensitivity tests with alternative consolidation distances (ε = 20 m and 40 m) likewise showed consistent declines in the most recent period (−44.4% and −40.0% declines, respectively).

### 3.2. Distribution Patterns and Grid-Cell Change

**Figure 2:**
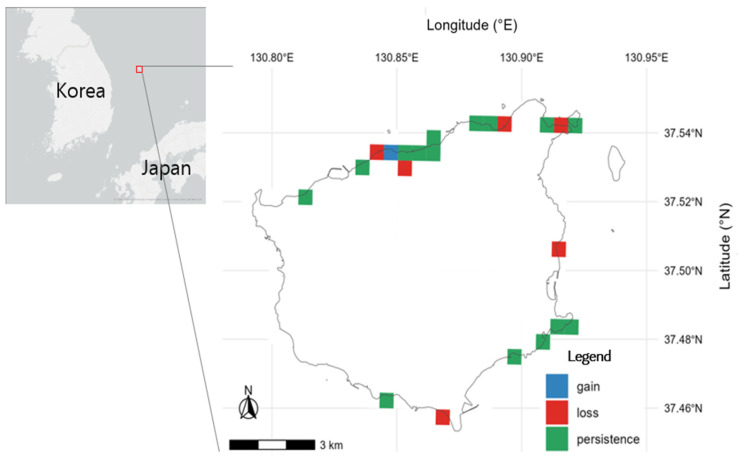
Grid-based status (500 m) relative to 2023–2024. Persistence: present in 2008–2022 and 2023–2024; Loss: present only in 2008–2022; Gain: present only in 2023–2024; No detection: absent in both periods. gain 1, loss 6, persistence 15.

A 500 m grid analysis of distributional change identified six loss cells in 2023–2024 (Figure 2): four along the northern coast, one on the eastern coast, and one on the southern coast. Persistence cells were concentrated along the northern–northeastern shoreline and parts of the south and east, whereas gain cells occurred on the northern coast. Both persistence and gain tended to cluster along mountain–cliff margins (e.g., stone revetments, slope edges and areas behind rockfall barriers), with some observations on gravel beaches. A pronounced spatial asymmetry emerged: continuous distributions along the northern and northeastern coasts contrasted with a scattered, fragmented pattern in the south and east. In the latter, larger gaps between occupied cells and intervening loss cells indicate reduced connectivity. Illustrative cases include the Songgot-san site on the north coast, which has not been reconfirmed since 2014; additional sites near coastal roads and certain beaches have also been lost in recent surveys, and one eastern site has field notes indicating competitive suppression by other vegetation.

### 3.3. Species Distribution Model (SDM) Results

**Figure 3:**
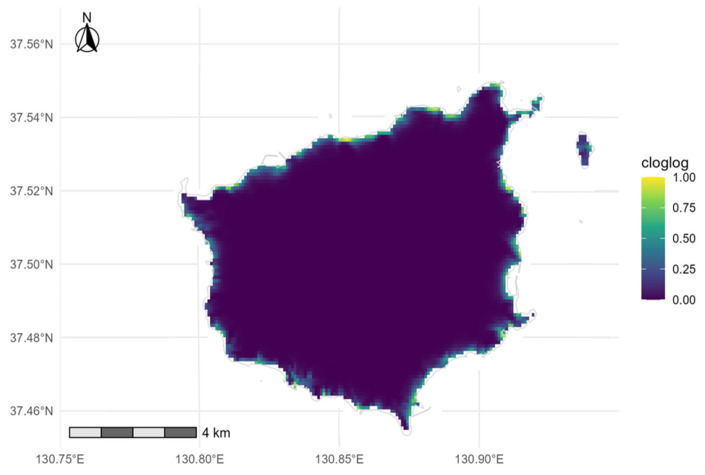
MaxEnt cloglog suitability (5 m) for *S. takesimensis* on Ulleungdo. Model: lq features, RM = 1.40, train 2020–2024 (unique presences n = 28; background n = 744).

Using *maxnet* with linear–quadratic (lq) features and an accessible background constrained to within 300 m from the shoreline, we fitted 28 unique presences against 744 background points (realized ratio: 26.6:1; target approximately: 25:1) and selected RM = 1.40. Cluster-based spatial holdout (leave-one-cluster-out; DBSCAN ε = 250 m) yielded LOCO AUC = 0.984 ± 0.014 and pAUC at a specificity of at least 0.90 was 0.935, with RelRMSE = 0.107, indicating strong spatial discrimination. The performance remained similar across clustering scales (DBSCAN ε = 150–300 m; AUC: 0.984–0.988; pAUC: 0.931–0.939; Table A1). MaxEnt cloglog predictions delineated a narrow, near-coastal high-suitability belt with a continuous northern core and fragmented southern–eastern satellites (Figure 3).

**Figure 4:**
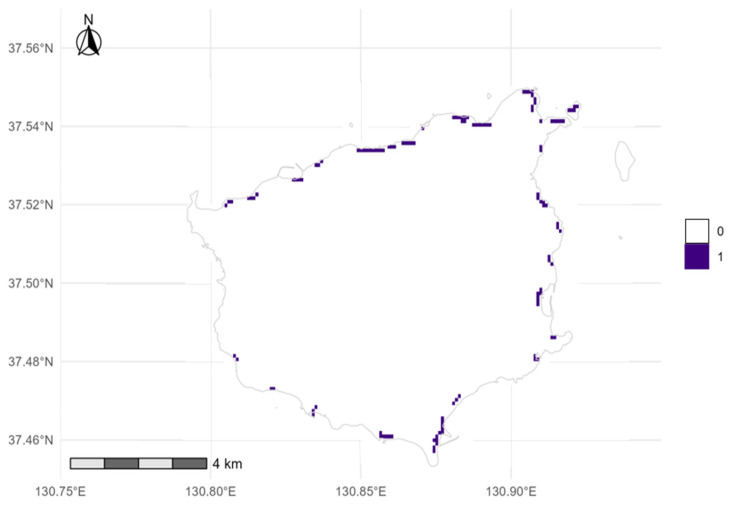
CORE habitat after post-processing (cloglog at or above 0.472, within 90 m of the coastline, and contiguous patches of at least three cells). Cells are coded 0 (non-core) and 1 (core). There was no leakage beyond the 90 m belt after post-processing (by design). Spec0.980 denotes the 98th percentile of background predictions from the training background sample.

For management, we adopted Spec0.980 (threshold = 0.472; baseline: 300 m) together with a 90 m coastal belt and a minimum patch size of at least three cells (75 m^2^), defining a CORE of 1.148 km^2^ with 71.4% recent presence coverage and, after post-processing, zero leakage outside the belt (Figure 4). In background-ratio sweeps under random CV (not LOCO), mean AUC was approximately 0.923–0.925 and pAUC approximately 0.802–0.810 (Table A2); these are not directly comparable to the LOCO AUC above due to different validation schemes. This highlights the expected precision–area trade-off as thresholds become more conservative (pre-policy areas: 3.728, 2.321, 1.860, and 1.763 km^2^ for Balanced, TP10, coverage-target, and Spec0.980, respectively). Additionally, at MaxTSS and BA (threshold = 0.322), the pre-policy area was 2.928 km^2^ with coverage = 0.929, but leakage outside the 90 m belt rose to 0.639 km^2^ and both PPV and F1 decreased to 0.636 and 0.778, relative to the corresponding Spec0.980 values (0.774 and 0.814), reinforcing our conservative choice (Figure A1). Estimated thresholds on the cloglog scale were Balanced (apparent) = 0.239, TP10 = 0.382, Coverage-target = 0.455, and Spec0.980 = 0.472 (Figure 4).

Sensitivity to the accessible background: Expanding the coastal background mask and resampling under the same design preserved the continuous northern–northeastern core and fragmented southeastern mosaics (Figure 3 and Figure 4). Under Spec0.980 with post-processing, the results were as follows: 300 m (thr 0.494; CORE 1.108 km^2^; coverage 0.714), 450 m (thr 0.380; 1.593 km^2^; 0.789), and 600 m (thr 0.522; 0.930 km^2^; 0.607). Across masks, the post-processed CORE spanned from 0.930 to 1.593 km^2^ (a spread of 0.663 km^2^; a 71.3% increase relative to the smallest extent), with coverage from 0.607 to 0.789; we recommend carrying this ‘area band’ forward in planning.

**Figure 5:**
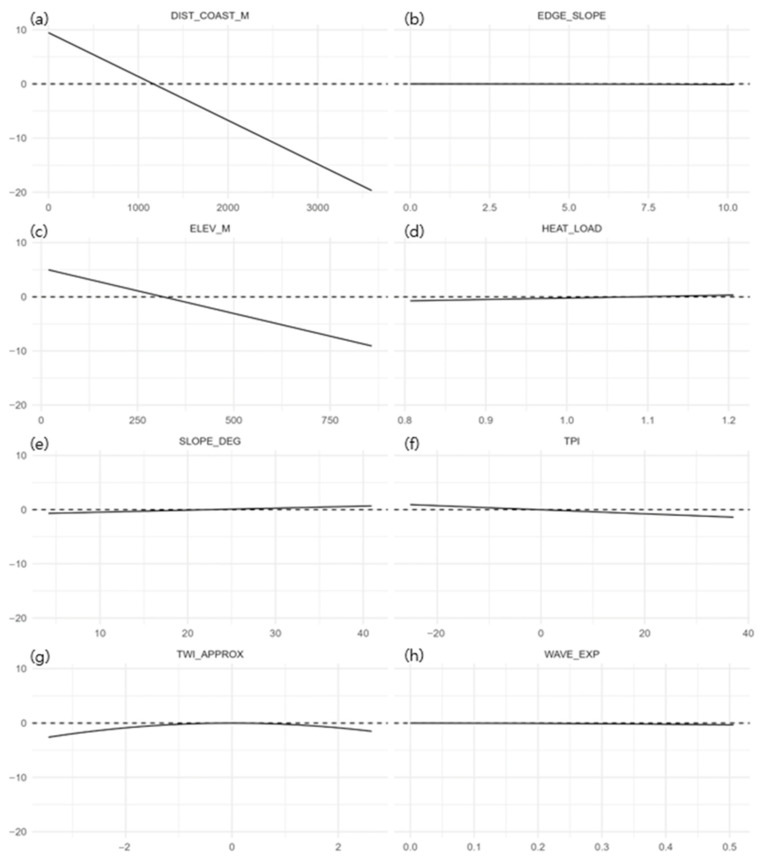
Partial response (Δ-link) curves for the eight most influential predictors of *Scrophularia takesimensis* suitability on Ulleungdo: (**a**) distance to coast (m), (**b**) edge slope, (**c**) elevation (m), (**d**) heat-load index, (**e**) slope (°), (**f**) topographic position index (TPI), (**g**) topographic wetness (approx.), and (**h**) wave exposure. Each panel shows the change in the model’s linear predictor relative to median conditions while only varying the focal predictor; the dashed line denotes no effect.

In partial response (Δ-link) curves, the distance to coast (DIST_COAST_M) and elevation (ELEV_M) showed negative effects, indicating increasing suitability at low elevations close to the shore (Figure 5). TPI displayed a weak negative tendency; SLOPE_DEG showed a gentle convex response (avoidance of overly steep slopes); and TWI_APPROX showed a mild unimodal peak (preference for intermediate wetness). The contributions of other predictors were comparatively small.

## 4. Discussion

### 4.1. Range Contraction and Underlying Threats

Long-term monitoring indicates recent losses in several grid cells. Given that period-specific detection rates remained relatively stable (0.65–0.75), these losses are unlikely to be an artifact of variable survey effort and instead suggest true range contraction. These losses are consistent with our spatially explicit validation (LOCO), which supports that the pattern is not an artifact of model reuse or sampling idiosyncrasies. Nonetheless, residual heterogeneity in detection probability (e.g., short-term weather, observer effects, and site accessibility) may persist; accordingly, we interpret the magnitude of contraction conservatively and rely on convergent evidence (stable detection rates with overlapping bootstrap CIs, consistent grid-cell losses, and field notes) to support a real decline. Chronic disturbance along the coastal interface—road widening, tourism development, slope engineering, and competitive suppression by co-occurring vegetation—has been flagged as a major pressure and is consistent with our field notes for multiple lost sites. In insular endemics, local habitat loss can erode genetic diversity and compromise adaptive capacity over time [50]. Accordingly, maintaining the continuous northern–northeastern core and restoring connectivity across the fragmented southern–eastern sector are urgent priorities.

### 4.2. Spatial Asymmetry and Ecological Implications

Our analyses reveal a pronounced spatial asymmetry: a continuous northern core contrasted with fragmented southern and eastern satellites (Figure 2). Although small, isolated satellites on islands are generally prone to genetic bottlenecks and inbreeding [50,51], our inferences about gene flow and demographic exchange between the northern and southern–eastern groups are based on spatial patterns rather than direct evidence. A recent master’s thesis provides initial population-genetic information for *S. takesimensis* [52], but island-wide, peer-reviewed analyses are not yet available; therefore, we treat statements about connectivity and diversity as provisional. Accordingly, we refrain from prescriptive claims about genetic exchange and present these as testable hypotheses to be evaluated with island-wide sampling and formal analyses (e.g., population genetics and connectivity modeling). In short, the genetic concerns for the southern and eastern groups are ecologically plausible, but remain hypothetical pending comprehensive genetic data.

The continuity of northern coastal populations suggests relatively unobstructed gene flow and may confer a greater capacity for recovery through demographic exchange after local disturbances. By contrast, the southeastern satellite populations are more isolated and smaller, making them more vulnerable to local extinction, particularly under minor habitat degradation or competitive exclusion. This pattern is consistent with studies reporting genetic structuring and potential diversity loss in fragmented habitats [53]. However, these inferences are based on spatial patterns rather than direct genetic evidence; targeted, island-wide genetic sampling is needed to verify connectivity and diversity outcomes.

While short-term demographic persistence may be possible in these isolated populations, prolonged isolation can diminish the species’ adaptive potential under environmental change. Historical records show repeated loss-gain along the northern coast, whereas losses outweighed gains in the east [54]. For the southeastern populations, we recommend microhabitat provisioning (e.g., windbreaks, nurse shrubs, debris retention), small-scale reinforcement translocations to preserve genetic diversity, and strategically placed stepping-stone habitats to restore connectivity and alleviate genetic bottlenecks. To avoid genetic homogenization, reinforcement should follow seed-zone or family-structured designs once the baseline genetic structure is resolved. These measures aim to improve population viability by enhancing gene flow and genetic resilience.

We propose to evaluate functional connectivity using least-cost paths and circuit theory models parameterized with our SDM outputs and coastal geomorphology. We plan to conduct population genetic sampling along the north–south gradient to quantify gene flow and assess connectivity among fragmented populations. Although hydrochory (alongshore passive transport) is plausible [18], it remains unproven; we will consider employing seed and propagule tracking and genetic assignment tests to verify dispersal mechanisms. We will transparently document cost surface construction (e.g., one minus cloglog suitability, slope penalties) and parameter choices to support reproducibility. We strongly recommend that future work include expanded genetic sampling to validate assumptions about gene flow and diversity loss, thereby improving inference on connectivity and genetic resilience.

### 4.3. Interpretation of the SDM and Methodological Considerations

We explicitly prioritized minimizing false positives for management and adopted the Spec0.980 threshold (0.472; Table A3). Relative to the Balanced (apparent) criterion (3.728 km^2^), this choice reduced the pre-policy mapped area to 1.763 km^2^ while increasing specificity to 0.980 and PPV to 0.774. Combining this threshold with a 90 m coastal belt constraint and a minimum-patch filter of at least 3 cells (75 m^2^) yielded a CORE of 1.148 km^2^ that captured 71.4% of recent observations, with no leakage beyond the coastal belt after post-processing (Figure 3 and Figure 4). Note that ‘no leakage beyond the 90 m belt’ is by construction after post-processing and must not be interpreted as ecological absence beyond the belt. Model performance remained high (LOCO AUC = 0.984 ± 0.014; RelRMSE = 0.107), indicating that, with small samples, using lq features with moderate regularization (RM = 1.40) and a conservative binarization scheme can effectively constrain overfitting. AUC and pAUC values from the background-ratio sweeps (Table A2) were obtained under random cross-validation and are not directly comparable to LOCO; we therefore treat LOCO metrics as the primary evidence of generalization. Despite strong cluster-holdout performance, small-n presence–background models may still be ecologically overfitted even when geographically transferable; response shapes for individual predictors should be read as conditional patterns rather than causal effects.

Ecologically, the strongest negative responses of DIST_COAST_M and ELEV_M indicate reliance on the narrow coastal–forest ecotone, where salt spray, high humidity, and thermal buffering co-occur on trachyte substrates. The shallow, convex response of SLOPE_DEG suggests avoidance of very steep, failure-prone faces while retaining moderate inclines that provide crevices and ledges; the mild unimodal TWI_APPROX response is consistent with a preference for intermediate drainage that prevents both desiccation and prolonged waterlogging. Together, these patterns point to a mechanistic niche shaped by maritime exposure and cliff-edge microtopography rather than macroclimate.

Comparable coastal-cliff endemics on oceanic islands show similar confinement to near-shore belts, coupled with salt-spray tolerance and sensitivity to coastal engineering (e.g., cliff stabilization, revetments). Our pattern—a continuous, wind-exposed core with fragmented lee-side satellites—mirrors cases reported for other island chasmophytes, where exposure gradients and access infrastructure jointly structure occupancy [55]. We therefore interpret *S. takesimensis* as following a general maritime-ecotone syndrome rather than exhibiting an idiosyncratic response.

Methodological choices that improved specificity (Spec0.980, together with the coastal-belt filter and MMU filter) are not merely statistical; they operationalize the inferred ecology by privileging patches with sustained maritime exposure and sufficient microhabitat extent, thereby translating niche signals into defensible spatial prescriptions. Because our predictors are topographic and exposure proxies, these responses should be read as mechanistic hypotheses—salt spray, substrate chemistry, and boundary-layer microclimate—to be tested with targeted soil chemistry and microclimate logging along coast-to-interior transects.

Sensitivity to the accessible background: Expanding the coastal background mask and resampling under the same design preserved the northern–northeastern axis and the fragmented southeastern mosaics (Figure 3 and Figure 4). Under Spec0.980 with post-processing, the results were as follows: 300 m (threshold 0.494; CORE 1.108 km^2^; coverage 0.714), 450 m (threshold 0.380; CORE 1.593 km^2^; coverage 0.789), and 600 m (threshold 0.522; CORE 0.930 km^2^; coverage 0.607; Table 1). We retain the within-300 m background as the ecological baseline because it best approximates the accessible coastal ecotone and the survey footprint (Q90: the 90th percentile of coastal distance, approximately 90 m). The 450 m and 600 m runs are treated as stress tests that bound uncertainty rather than co-equal models. Accordingly, we carry forward an area band of 0.930–1.593 km^2^ for planning to represent mask-driven uncertainty explicitly.

In practice, we adopt a tiered, trigger-based plan: Tier-1 (no-regrets) is the adopted CORE (1.148 km^2^). Tier-2 (contingency) extends to polygons consistent with the 450 m mask, activated only after (i) at least two seasonal re-detections or (ii) a habitat-quality audit (substrate, salt spray, slope stability). Tier-3 (survey-first) only targets the areas present under the 450 m or 600 m masks. Under post-processing, MaxTSS and BA introduced substantial belt-external leakage before belt clipping (approximately 0.64 km^2^ with a 90 m belt and MMU of 3; Figure A2), whereas Spec0.980 showed small pre-belt leakage (approximately 0.2 km^2^) and zero after applying the belt filter; therefore, we adopted Spec0.980 for regulation-ready products. Because PPV and NPV are computed relative to background (pseudo-absences), they should not be interpreted as absolute probabilities of field absence or presence.

Ecological interpretation: Δ-link responses indicated negative effects of DIST_COAST_M and ELEV_M; SLOPE_DEG showed a shallow, convex response consistent with avoidance of overly steep terrain, and TWI_APPROX exhibited a mild unimodal peak indicating preference for intermediate wetness. The limited added value from WAVE_EXP and EDGE_SLOPE likely reflects two factors: (i) coastal distance already subsumes much of the coastal signal, and (ii) the assumed decay lengths (150–300 m) may not match the operative environmental scale. Together with the belt-constrained CORE behavior, these patterns support genuine reliance on the coastal forest ecotone rather than artifacts of thresholding.

Although we did not assay soils or microclimate in situ, the confinement of occurrences to coastal ecotones on trachyte-derived substrates suggests that edaphic filters (drainage, base cation inputs from marine aerosols, and relative alkalinity) interact with oceanic microclimate and topographic exposure to shape the realized niche of S. *takesimensis* [12,56]. This interpretation is consistent with the negative Δ-link effects of DIST_COAST_M and ELEV_M and the mild unimodal TWI_APPROX response reported above. Future work integrating soil chemistry (pH, CEC), texture, and microclimate logging across slope aspects and cliff-interior transects will be critical to test these mechanisms and to refine conservation prescriptions. In line with our variable design, we interpret DIST_COAST_M and ELEV_M primarily as proxies for a coastal microclimate and exposure gradient (salt spray, humidity, thermal buffering), rather than as singular causal factors. This proxy interpretation also explains why we do not extend these results to future climates, given the static predictor set.

### 4.4. Data-Driven Spatial Conservation Strategy

Using accessible background masks of 300, 450, and 600 m—which bound the post-processed CORE between 0.930 and 1.593 km^2^—we adopt a tiered plan: Tier-1 (adopted CORE, 1.148 km^2^); Tier-2 (conditional expansion), activated only after at least two seasonal repeat detections or a habitat-quality audit; and Tier-3 (survey-first) targeting areas present only under the wider masks.

(1) Core Conservation Zone: Recognize the continuous northern–northeastern coastal belt as the central conservation axis. Designate the northern CORE as a strict protection area, prioritize safeguarding habitat integrity, and minimize anthropogenic disturbance. Prioritizing the wind-exposed northern belt aligns with the species’ dependence on salt spray and edge microclimate and with the observed continuous core; minimizing revetment hardening and over-stabilization prevents the loss of crevice microhabitats implied as limiting by the SDM. Habitat damage should be minimized during road maintenance and slope stabilization, and—anticipating extreme rainfall—stability monitoring of habitats near steep slopes should be carried out in parallel using high-resolution LiDAR-based terrain-change analyses and drone (uncrewed aerial vehicle, UAV) surveys to detect signs of slope failure and soil erosion [57].

(2) Restoration Priority Zone: In the isolated, fragmented eastern and southern sectors, we focus on connectivity. In the fragmented southeastern sector, short-gap connectivity measures should target demographic rescue and pollination continuity in lee-side areas; small, diversity-conscious reinforcements can reduce inbreeding risk until natural exchange is re-established. Mitigate fragmentation-driven declines in effective population size and adaptive capacity [58] through competitive vegetation control; conduct microhabitat enhancement (e.g., rock-crevice niches) to promote flowering and pollination; and, where justified, conduct small-scale diversity-conscious reinforcement translocations to establish stepping-stone habitats [59]. The objective is not merely to increase abundance, but to alleviate bottlenecks and sustain long-term adaptive potential. Interventions should proceed under an adaptive framework that updates priorities as connectivity and genetic results accumulate. Where practicable, interventions are triggered only after at least two seasonal re-detections or following a habitat-quality audit, consistent with Tier-2.

(3) Targeted surveys for new localities: To decouple suitability from detectability, rankings incorporate recent effort and safety constraints and geomorphically plausible niches (gravel and ledge systems) inferred from response shapes. We identified candidate survey segments by summarizing cloglog predictions along the coastline (rolling window) and selecting stretches where the segment-median suitability was at or above the Spec0.980 threshold and contiguity was maintained within the 90 m belt. We then adjusted ranks using (i) recent survey-effort gaps, (ii) access and safety, and (iii) microhabitat priors from the literature. Under this scheme, gravel beaches between Jeodong and Seommok form the Priority-1 segment because high-suitability predictions coincide with repeatedly reported substrate conditions. To avoid conflating suitability with detection probability, we report quantile-based priority bands rather than absolute discovery probabilities and explicitly note that discovery probabilities must be calibrated with future occupancy–detection data. These actions integrate with existing national frameworks (e.g., the Endangered Wildlife Nationwide Distribution Survey and NIE restoration planning), facilitating adoption without creating parallel processes. This creates a reproducible monitor–model–manage loop: standardized surveys feed SDM updates, which in turn refine priority tiers and field protocols.

## 5. Conclusions

Island-wide surveys and a fine-resolution species distribution model show a recent decline in unique sites and a strong spatial asymmetry for *Scrophularia takesimensis* (continuous, wind-exposed northern core versus fragmented southeastern satellites). A parsimonious MaxEnt model (lq; RM = 1.40; 28 presences; 744 background) generalized well under cluster holdout (LOCO AUC 0.984 ± 0.014; RelRMSE 0.107), and key predictors act as proxies for maritime exposure and cliff-edge microtopography. Using a conservative threshold (Spec0.980 = 0.472) with a 90 m coastal belt and a minimum patch of three 5 m cells produced a regulation-ready CORE of 1.148 km^2^ that included 71.4% of recent records, with no leakage beyond the belt after post-processing (by design). Mask sensitivity defines a planning area band of 0.930–1.593 km^2^; we operationalized this using a tiered framework: protect the adopted CORE (Tier-1), conditionally extend to polygons consistent with the 450 m run upon predefined triggers (at least two seasonal re-detections or a habitat-quality audit) (Tier-2), and target surveys to segments that appear only under wider masks (Tier-3). Because predictors are static topographic and exposure proxies, inferences concern present-day suitability rather than future climates; priorities now are soil chemistry and microclimate logging, island-wide genetics and connectivity modeling, and continued monitoring to refine thresholds and tiers.

## Figures and Tables

**Table 1:** Post-processed CORE under Spec0.980 across accessible background masks (with a 90 m coastal belt and contiguous patches of at least three cells).

Background (m)	Spec0.980_thr	CORE_Area (km^2^)	Coverage_Recent
300	0.494	1.108	0.714
450	0.380	1.593	0.789
600	0.522	0.930	0.607

Note. Spec0.980 is computed as the 0.98 quantile of background predictions from each run’s training background sample; therefore, the numeric thresholds differ across masks and sweeps. The adopted regulation-ready core uses 0.472 from the baseline 300 m run, whereas the Table 1 300 m entry (0.494) comes from the mask-sweep resampling.

## Data Availability

We do not release any coordinates publicly—neither precise localities nor 500 m grid centroids—under endangered-species policy and permit conditions. On a narrow coastal ecotone, even 500 m cells linked with published site lists or high-resolution basemaps can enable linkage attacks; thus, only aggregate, non-locator statistics are shared. Analysis code is openly available as Code S1 (public-safe, version-locked, fixed seeds). The repository contains no locality coordinates and blocks map-like exports in PUBLIC_MODE, enabling a full procedural audit without risking sensitive locations. These materials allow for full computational reproducibility without exposing sensitive coordinates. Controlled access to coordinates may be granted to qualified researchers for non-commercial conservation research under a Data Use Agreement (no redistribution; secure disposal at project end) and with approval from the competent authority.

## Supplementary Materials

The following supporting information can be downloaded at: https://www.mdpi.com/article/10.3390/plants14223498/s1, Code S1, Reproducible R scripts and parameter settings for species distribution modeling and spatial analyses of *Scrophularia takesimensis* on Ulleungdo.

## Appendix A

**Table A1:** LOCO performance across DBSCAN epsilon values used to define cluster folds. Cluster folds were defined by DBSCAN with minPts set to 1, and the modeling recipe (linear and quadratic features, RM 1.40, same background design) was held constant. Partial AUC denotes the standardized partial AUC computed over a false positive rate of 0.10 or less.

ε (m)	n_clusters (Folds)	AUC (Mean ± SD)	pAUC (Mean ± SD)
150	19	0.988 ± 0.014	0.939 ± 0.071
200	17	0.987 ± 0.014	0.932 ± 0.073
250	15	0.984 ± 0.014	0.935 ± 0.074
300	14	0.987 ± 0.014	0.931 ± 0.076

**Table A2:** Background-ratio and accessible-mask sensitivity: (a) auc_mean, (b) auc_sd, (c) pAUC_mean, (d) thr_bal_med, (e) spec_at_thr, (f) thr_cov_med, (g) proportion of island area, (h) area_spec_sd, (i) cov_spec_mean. These AUC and partial AUC values were obtained under random cross-validation during the ratio and mask sweeps and are not directly comparable to the LOCO metrics.

Ratio	n	(a)	(b)	(c)	(d)	(e)	(f)	(g)	(h)	(i)
10	40	0.925	0.010	0.810	0.319	0.933	0.019	0.020	0.019	0.057
15	40	0.925	0.007	0.808	0.294	0.950	0.013	0.023	0.013	0.070
20	40	0.924	0.006	0.805	0.304	0.957	0.009	0.018	0.012	0.054
25	40	0.925	0.005	0.807	0.302	0.966	0.007	0.023	0.014	0.068
30	40	0.923	0.004	0.802	0.286	0.971	0.007	0.024	0.008	0.073

**Table A3:** Pre-policy threshold comparison on cloglog predictions (adopted rule: Spec0.980).

Threshold	Thr	Pred. Area (km^2^)	Sens	Spec	PPV	NPV	Note
Balanced (apparent)	0.239	3.728	1.000	0.946	0.412	1.000	Pre-policy (median Youden across folds)
TP10	0.382	2.321	0.893	0.970	0.532	0.996	Pre-policy
Coverage-target	0.455	1.860	0.857	0.979	0.600	0.995	Pre-policy (coverage at least 0.90)
Spec0.980	0.472	1.763	0.786	0.980	0.774	0.992	Adopted (Pre-policy)

Note. Balanced (apparent): single-model Youden on the full training set. Balanced-CV: median Youden across CV folds (used for tuning only).
